# Biological Activities of *Galanthus fosteri* Extracts: First Demonstration of the Interaction between Chlorogenic Acid and DNA Ligase by Molecular Docking

**DOI:** 10.1021/acsomega.4c00162

**Published:** 2024-02-23

**Authors:** Safiye Elif Korcan, Nevin Çankaya, İbrahim Bulduk, Gencer Güvercin, Şah İsmail Çivi

**Affiliations:** † Vocational School of Health Services, Uşak University, Uşak 64200, Turkey; ‡ Faculty of Engineering, Department of Chemical Engineering, Afyon Kocatepe University, Afyonkarahisar 03200, Turkey; § Department of Bioengineering, Yeditepe University, İstanbul 34755, Turkey; ∥ Faculty of Engineering and Natural Sciences, Department of Molecular Biology and Genetics, Uşak University, Uşak 64200, Turkey

**Keywords:** *Galanthus fosteri*, biological activity, antioxidant, antimicrobial, chlorogenic acid, DNA ligase

## Abstract

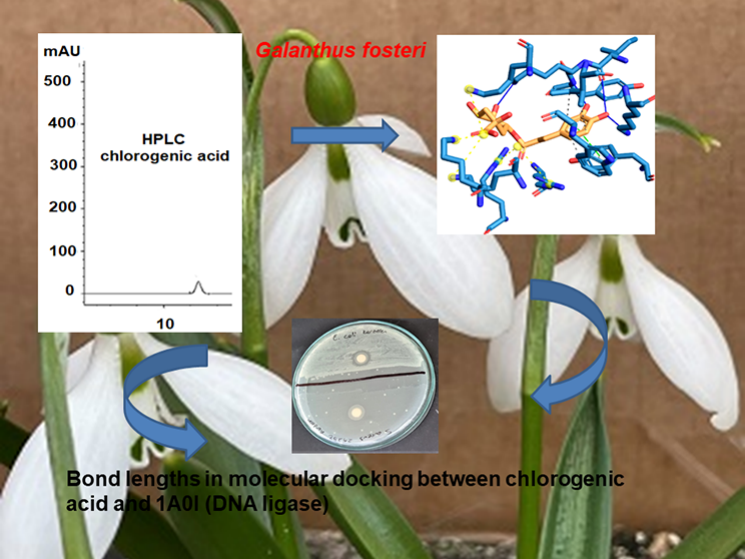

Within the Amaryllidaceae family, the bulbous plant species *Galanthus fosteri* (*G. fosteri*) belongs to the *Galanthus* genus. Alkaloids with a broad variety of biological functions are typically found in the flora of this family. The *G. fosteri* plant’s organs’ antioxidant activity, antibacterial impact, and antimicrobial qualities were examined in this study. Total flavonoid contents (TFC) and total phenolic contents (TPC) of plant extracts were measured with spectrophotometric methods, and antioxidant activity was determined using the DPPH (2,2-diphenyl-1-picrylhydrazyl) radical scavenging technique. The HPLC method was used to determine the phenolic compounds on a component basis. The antibacterial properties of the extracts were assessed using the Kirby−Bauer disc diffusion method, and the minimum inhibitory concentration method against the pathogens *Klebsiella pneumoniae*, *Staphylococcus aureus*, *Pseudomonas aeruginosa*, and *Escherichia coli*. Additionally, combination tests were performed between the extract and antibiotics. Leaf and stem extracts demonstrated greater antioxidant qualities than bulb extracts, despite the fact that extracts of plant organs did not exhibit appreciable levels of TPC, TFC, or antioxidant qualities. According to the HPLC analysis results, it was determined that chlorogenic acid was present in all of the extracts. In fact, it was determined that only chlorogenic acid was 8.02 (mg/10 g) in *G. fosteri* bulb peel, which has antimicrobial and antioxidant properties. A molecular docking study has demonstrated for the first time that the antibacterial effect of chlorogenic acid might be due to DNA replication inhibition.

## 1 Introduction

Many *Galanthus* spp. are distributed around Anatolia, the Caucasus, Thrace, the Eastern Mediterranean, and the Black Sea region. There are different types.^1,2^ Although *Galanthus* sp. is used as an ornamental plant, alkaloids such as galantamine, especially found in its bulbs, are of economic importance.

*Galanthus fosteri* (*G. fosteri*), distributed in southern and north-central Anatolia, is a monocotyledonous bulbous plant that is a member of the Amaryllidaceae family.^3^ It has been reported by many researchers that the phenolic, flavonoid, and alkaloid-rich extracts of these family members have antioxidant, antimalarial, hepatoprotective, antitumor, anti-inflammatory, and antiviral effects.^4−10^ Phenolic compounds are important antibacterial substances that are known to have antioxidant activity. Nevertheless, phenolic compounds’ antibacterial activity mechanisms remain incompletely understood. These compounds show multiple effects at the cellular level.^11^ Numerous investigations have revealed that phenolic compounds have the power to alter the permeability of the cell membrane, induce intracellular functions of enzymes by binding to enzymes with hydrogen bonds, and cause disruption of the integrity of the cell wall.^12−16^ For example, phenylpropanoids and tannins can cause damage to the cell membrane or inhibit metabolic enzymes by binding to them.^17,18^ Phenolic chemicals are lipophilic, which makes it easier for them to interact with cell membranes and boosts their antibacterial activity.^19^ By attaching to soluble extracellular proteins and the bacterial cell wall, flavonoids create complexes.^15,20^ They also inhibit both energy metabolism and DNA and RNA synthesis in the bacterial cell.^21^ They can affect ATP production as well as intracellular pH modification in Gram-positive bacteria.^22^

Nowadays, the use of alternative antimicrobial and antioxidant herbal sources simultaneously while antibiotic treatment continues is common among the public. However, there are not enough studies on whether the natural resources used have a synergistic or antagonistic effect on antibiotics. In most studies conducted to date, this situation has been ignored, and only the biological activities of plant extracts have been investigated. In this study, after the antioxidant and antimicrobial effects of *G. fosteri* water and alcohol extracts were evaluated, combination tests of the extract with some frequently used antibiotics were performed.

## 2 Materials and Methods

Species identification of the samples collected from Turkey’s Samsun province (36 E 20 and 41 N 17) in February 2021 was made using the web address http://194.27.225.161/yasin/tubives/index.php. The purity level of all reagents used in the antioxidant study was ≥99% and was obtained from Sigma Chemical Co. Bacteria used to determine antibacterial properties (*Escherichia coli* ATCC 35213, *Staphylococcus aureus* ATCC 25292, *Pseudomonas aeruginosa* ATCC 11778, and *Klebsiella pneumoniae* NRRL B 4420) were obtained from the Usak University Vocational School of Health Services Laboratory.

### 2.1 Ultrasound-Assisted Extraction

The leaves, stem, bulb, and root parts of *G. fosteri* were dried separately in a dark room at 27 °C ± 2 for 15 days. It was ground to a grain size of 80 mesh and ground in a mill. The dried, ground plant material (500 mg) was extracted with 100 mL of solvent (70% methanol + 30% pure water) in a WiseClean brand ultrasonic bath at a frequency of 50 kHz for 15 min. The white band filter paper (Whatman) was used to filter the extract, which was then refrigerated at +4 °C until analysis.

### 2.2 Determination of Total Phenolic Content and Total Flavonoid Content

Total phenolic content (TPC) in extracts was determined using the Folin−Ciocalteu reagent.^23,24^ Absorbance values were measured on a spectrophotometer (UV-1800 Shimadzu) at a wavelength of 765 nm. TPC is given as gallic acid equivalent (mg GAE/g). Using the aluminum chloride colorimetric approach, the extracts’ total flavonoid content (TFC) was assessed.^24,25^ Absorbance values were measured on a spectrophotometer device at a wavelength of 510 nm, and TFC was given as catechin equivalent (mg CAE/g).^24,25^

### 2.3 Determination of Phenolic and Flavonoid Substances by HPLC

The amounts of gallic acid, protocatechuic acid, chlorogenic acid, vanillic acid, syringic acid, caffeic acid, coumaric acid, ferulic acid, sinapinic acid, quercetin, and galantamine in methanol and water extracts were determined by HPLC. Using an Agilent brand 1260 HPLC with a UV detector, phenolic chemicals were detected. A 4.6 mm, 150 mm, 5 m ACE-C18 column was employed in the chromatographic separation process. At 1.0 mL min^−1^, the mobile phase flow rate was maintained constant. Ultrapure water with 0.1% acetic acid is mobile phase A; acetonitrile with 0.1% acetic acid is mobile phase B. The following are the gradient conditions: 0−3.25 min, 8−10% B; 3.25−8 min, 10−12% B; 8−15 min, 12−25% B; 15−15.8 min, 25−30% B; 15.8−25 min, 30−90% B; 25−25.4 min, 90−100% B; and 25.4−30 min, 100% B.

The column temperature was maintained at 25 °C, and the injection volume was 10 μL. The wavelengths at which the phenolic compounds under study have their highest absorption were taken into consideration when selecting the detection wavelengths. At 280 nm, syringic acid, protocatechuic acid, and gallic acid have been detected. At 225 nm, vanillic acid was identified. At 305 nm, coumaric acid has been identified. At 330 nm, caffeine and chlorogenic acid were recognized.^24^

### 2.4 Determination of the Antioxidant Effect by DPPH (2,2′-Diphenyl-1-picrylhydrazyl) Radical Analysis

In a 10 mL test tube, 300 μL of the sample extract and 5700 μL of the DPPH solution were combined. The combination was left to stand at room temperature in a dark area for 60 min. The Shimadzu UV-1800 spectrophotometer was used to measure the absorbance of the reaction mixture at 517 nm. Conversely, a control solution devoid of the sample extract was made, and its absorbance was assessed in comparison with ultrapure water. The antioxidant activity was calculated as follows:^23−25^
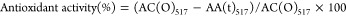


Here, AC(O)_517_ is the absorbance of the control at *t* = 0 min, and AA(t)_517_ is the absorbance of the antioxidant at *t* = 1 h. To compare the results obtained in the antioxidant test, an ascorbic acid solution at a concentration of 500 ppm was subjected to antioxidant activity testing under the same conditions.

### 2.5 Antimicrobial Activity Experiments

According to the HPLC analysis results, the amount of chlorogenic acid in *G. fosteri* bulb skin was determined to be 8.02 (mg/10 g). Additionally, since chlorogenic acid was the only compound detected among the compounds sought in *G. fosteri* bulb skin, this extract was used to determine the antimicrobial effect of chlorogenic acid.^26^

#### 2.5.1 Qualitative Determination of the Antibacterial Effect

Distilled water (100 mL) was added to 2 g of the ground extract. After being kept in a shaking oven at 125 rpm for 24 h, at 45 ± 5 °C, it was filtered using filter paper. The filtrate (20 μL) was absorbed into blank discs. The disc diffusion method was employed to ascertain the discs’ antibacterial efficacy. All experiments were repeated three times.

#### 2.5.2 Quantitative Analysis of the Antimicrobial Effect

The microdilution method was used to investigate the antimicrobial effects of *G. fosteri* bulbs. Sterile microdilution plates with 96 U-bottom wells were used for antimicrobial testing. Stock bacterial solutions at −80 °C were brought to room temperature, then inoculated into nutrient agar (NA) medium, and incubated at 37 °C for 24 h. Colonies formed after incubation in NA were planted in nutrient broth (NB) medium and incubated until they reached the 0.5 McFarland standard. The extracts (100 μL) at different concentrations and 100 μL of the bacterial culture were added to the wells. The minimum inhibitory concentration (MIC) value was determined after 24 h of incubation at 37 °C.^27^

#### 2.5.3 Antibiotic Combination Testing

In this method, empty antibiotic discs were impregnated with 20 of the determined MIC values of the plant extract and placed in the middle of the NA plate inoculated with a bacterial culture at 5 McFarland turbidity. Discs of the tested antibiotics were placed around it. After the plates were incubated at 37 °C for 18−24 h, synergy was determined by an increase in the diameter of the inhibition zone of at least 2 mm.^28^

### 2.6 Molecular Docking Analysis

The DNA ligase protein structure (PDB ID: 1A0I) was obtained from the Protein Data Bank (PDB). The file belonging to 1A0I was transferred to AutoDockTools. Water molecules were removed from the protein structure. The chemical structure of the chlorogenic acid (PubChem CID: 1794427) ligand, whose molecular structure is C_16_H_18_O_9_, was obtained from the National Library of Medicine/National Centre for Biotechnology Information. A molecular docking study was performed using AutoDock 4.1. Analyses and images were obtained with the Biovia Discovery Studio Visualizer 2021 program. The interaction of chlorogenic acid and 1A0I (DNA ligase) was simulated by using the BIOVIA Discovery Studio Visualizer software package.

### 2.7 Statistical Analysis

The difference between TPC, TFC, and DPPH% values was significant as determined by the Friedman test using the SPSS 28 Package program.

## 3 Results and Discussion

### 3.1 Phenolic and Flavonoid Content of *G. fosteri*

**Figure 1:**
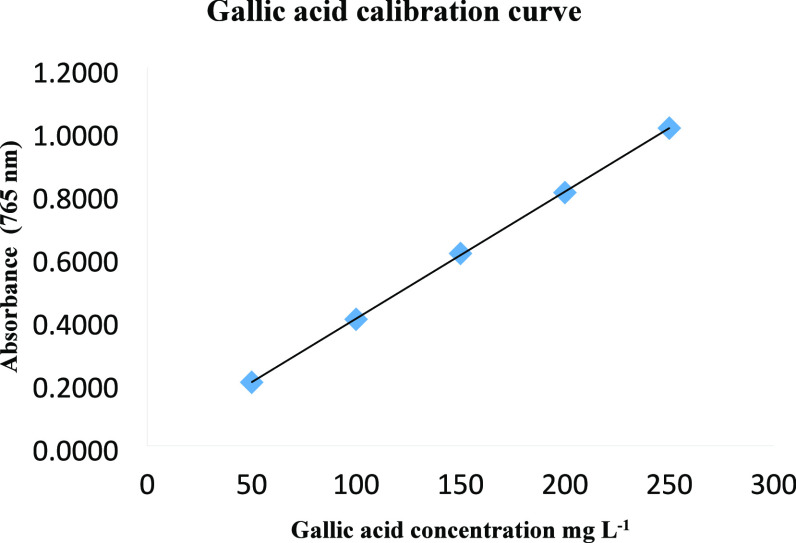
Gallic acid calibration curve.

A graph of absorbance values versus gallic acid concentrations was drawn and is presented in Figure 1. TPC was determined as mg GAE/g extract equivalents according to the equation *y* = 0.004*x* + 0.0001 obtained from the calibration chart. The correlation coefficient of the calibration curve was obtained as *R*^2^ = 0.9999.

**Figure 2:**
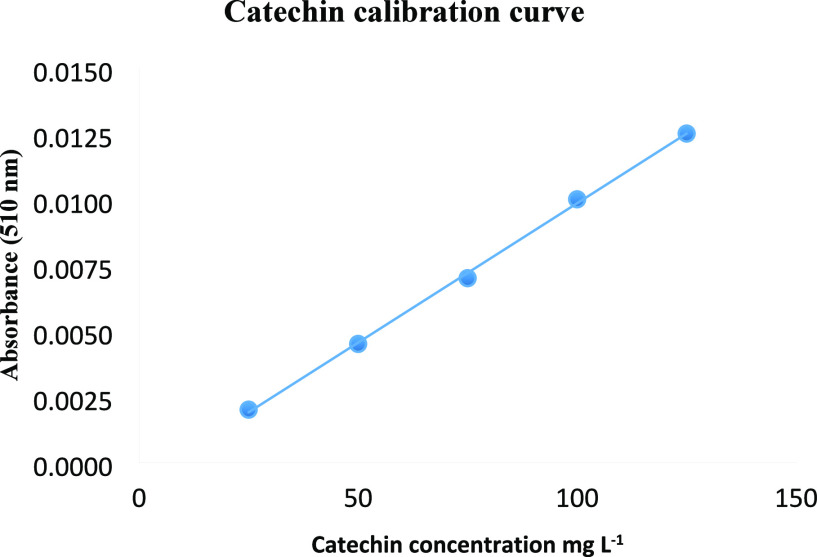
Catechin calibration curve.

TFC was measured as milligrams of CAE/g of extract equivalents according to the equation *y* = 0.0001*x* − 0.0007 obtained from the calibration chart. The correlation coefficient of the calibration curve was obtained as *R*^2^ = 0.9989. A graph consisting of absorbance values versus catechin concentrations was drawn and is presented in Figure 2.

**Table 1:** TPC, TFC, and DPPH % Inhibition Values in *G. fosteri* Methanol and Water Extracts^a^

*G. fosteri*	extract	TPC mg GAE/g sample	TFC mg CAE/g sample	inhibition%	Friedman test *p* value
leaf	methanol	16 ± 0.019	13 ± 0.001	80 ± 0.011	0.223
	water	19 ± 0.131	21 ± 0,003	34 ± 0.047	0.225
trunk	methanol	15 ± 0.035	18 ± 0.001	78 ± 0.045	0.135
	water	18 ± 0.145	20 ± 0.000	49 ± 0.05	0.135
bulb skin	methanol	13 ± 0.028	19 ± 0.002	45 ± 0.015	0.135
	water	9 ± 0.026	12 ± 0.002	74 ± 0.06	0.013*
bulb	methanol	13 ± 0.006	9 ± 0.002	45 ± 0.006	0.225
	water	12 ± 0.004	13 ± 0.001	22 ± 0.024	0.100
root	methanol	13 ± 0.012	12 ± 0.002	43 ± 0.029	0.135
	water	12 ± 0.033	9 ± 0.001	31 ± 0.053	0.013*

a *p < .05.

It was observed that the TPC values in *G. fosteri* methanol and water extracts varied between 9.0 mg GAE/g (bulb skin water extract) and 19.0 mg GAE/g (leaf water extract). It was determined that the most TPC was in the leaf water extract. The *G. fosteri* extracts seen in Table 1 are the leaf water extracts with the highest TFC content, with 21.0 mg of CAE/g sample. This result is similar to the TPC analysis result. This is followed by branch water extracts (20.0 mg CAE/g sample) and bulb skin methanol extracts (19.0 mg CAE/g sample). The amounts of galantamine determined in plant organs by the HPLC device of stock solutions prepared with water are given in milligrams per gram in Table 2 below. While it was observed that ascorbic acid solution (500 ppm) inhibited DPPH solution by 98.5%, the 80% inhibition value of methanolic extracts of plant leaves is a very satisfactory result. The Friedman test *p* value *p < .05, and the difference is significant. As a result of the Friedman test (nonparametric) performed in the SPSS 28 Package program, it was observed that there was a significant difference (*p* < .05) between the TPC, TFC, and inhibition values of *G. fosteri* root water extracts.

### 3.3 Determined Substance Amount by HPLC

**Figure 3:**
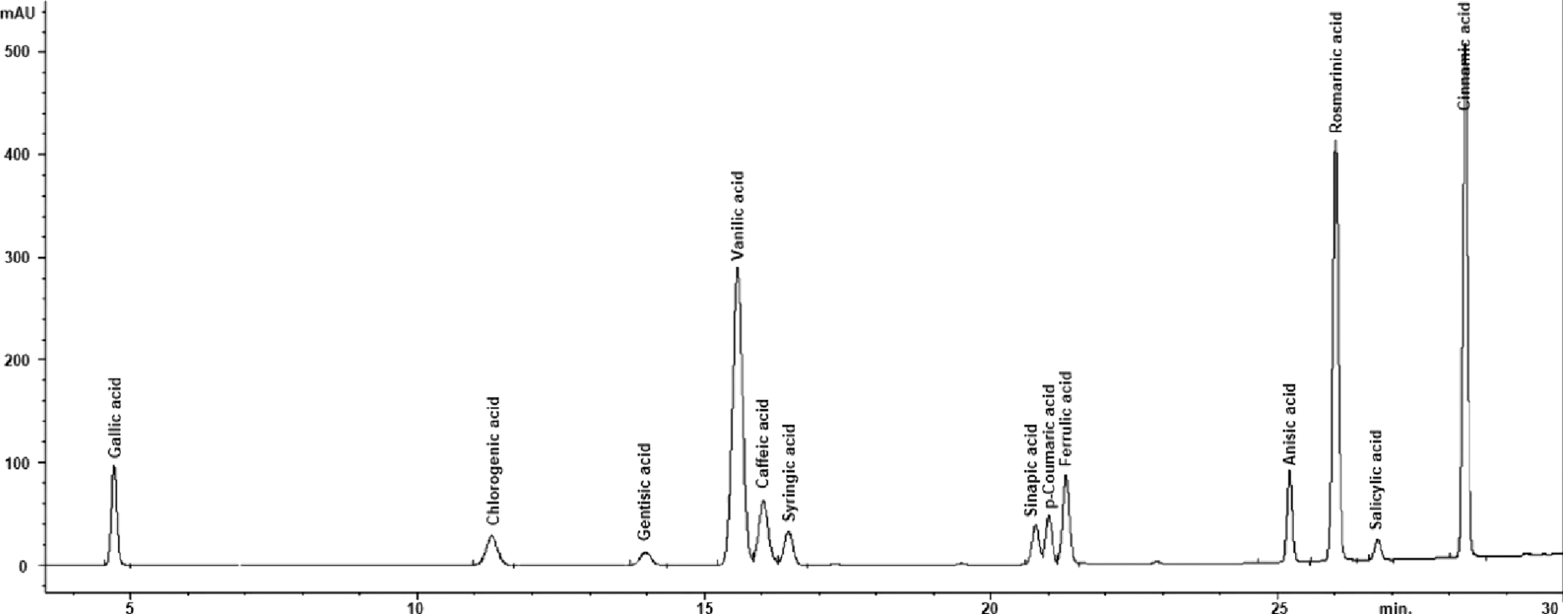
HPLC chromatogram of phenolic standards.

The substances detected in water and methanol extracts by the HPLC device are given in Table 2. In water extracts, quercetin (13.47 mg/10 g and 10.72 mg/10 g) in the leaf and root, galantamine (4.028 mg/10 g) in the stem, and chlorogenic acid (2.88 mg/10 g and 8.02 mg/10 g) in the bulb and bulb skin are the most abundant substances. The most abundant substance in methanol extracts is caffeic acid (11.2 mg/10 g) in the leaf and chlorogenic acid in other organs. The HPLC chromatogram of phenolic standards is given in Figure 3.

**Table 2:** HPLC Analysis Results of Extracts (mg/10 g)

	water extract					methanol extract				
	leaf	stem	bulb skin	bulb	root	leaf	stem	bulb skin	bulb	root
gallic acid									1.67	1.12
protocatechuic acid	5.70	2.12						1.22		
chlorogenic acid	5.00	6.06	8.02	2.88	5.38	6.66	10.36	2.76	2.12	2.82
vanillic acid				2.00		5.06			1.07	2.14
syringic acid					6.66					
caffeic acid	1.77	5.20				11.20	5.82			
coumaric acid						1.22	0.62	0.324		0.64
ferulic acid	0.29	0.56			1.05	3.20	1.02			
sinapic acid		1.54								1.80
quercetin	13.47				10.72	2.13				
galantamine	5.78	4.03		2.49						

**Table 3:** Antimicrobial Effect of *G. fosteri* Bulb Water Extracts and Combination Test Results^a^

	G. fosteri bulb water extract (0.4 mg/20 μL)		positive control inhibition zone (mm)					
microorganism	inhibition zone (mm)	MIC (μL/mg)	VA_30_	DA_2_	P_10_	OFX_5_	additive effect	synergistic effect
*E. coli* ATCC 35213	10	1.0000	14		8	46	VA, DA, P, OFX	
*S. aureus* ATCC 25292	13	0.0625	14		10	29	VA, DA, P	OFX
*P. aeruginosa* ATCC 11778	17	0.03125	25	11	8	37	VA, DA, P	OFX
*K. pneumoniae* NRRL B 4420	21	0.03125	15		13	44	VA, DA, P, OFX	

a VA, vancomycin; DA, clindamycin; P, penicillin; OFX, ofloxacin.

**Figure 4:**
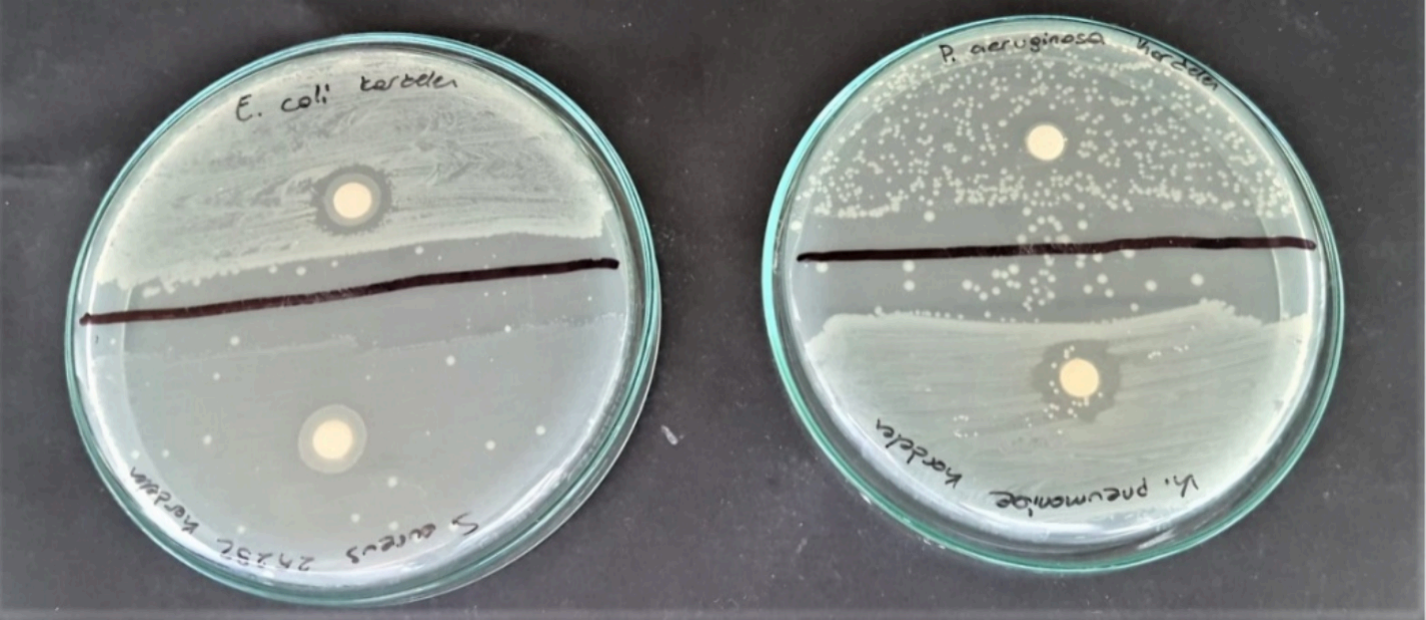
Antimicrobial effect of *G. fosteri* by the disc diffusion technique.

### 3.4 Free Radical Scavenging Effect

The DPPH% inhibition values of *G. fosteri* extracts are shown in Table 1. The maximum percentage of inhibition was determined to be 80 ± 0.011% in leaf methanol extracts. It was found that with the exception of bulb skin, methanol extracts’ percentage inhibition was higher than that of water extracts. The lowest inhibition was determined to be 22 ± 0.024% in bulb water extracts (Table 1). In this study, chlorogenic acid was the only component found in all extracts.Researchers are still interested in phenolic acids because of their diverse biological and pharmaceutical properties. Chlorogenic acid, one of the phenolic acids, is currently classified as caffeoylquinic acid isomers (3-, 4-), known as 5-CQA, according to the guidelines of the International Union of Pure and Applied Chemistry (IUPAC), and 5-CQA is the most abundant isomer. Chlorogenic acid is known to scavenge free radicals. Additionally, chlorogenic acid is a central nervous system stimulant and modulates lipid and glucose metabolism. For this reason, it is thought that it can help treat many diseases, such as hepatic steatosis, cardiovascular disease, diabetes, and obesity.^29^ Naveed et al. reported that mild centrizonal necrosis and Kupffer cell hyperplasia were observed as a result of chlorogenic acid treatment for hepatic necrosis. They attributed this to the synergistic antioxidant activity resulting from the binding of chlorogenic acid and lysozyme.

Similar to our results, it has been reported that chlorogenic acid scavenges superoxide radicals, hydroxyl radicals, and peroxynitrite under in vitro conditions in direct proportion to the concentration. Furthermore, research conducted in vivo has revealed that chlorogenic acid demonstrates a range of antioxidant actions in response to stomach mucosal injury generated by indomethacin.^30,31^ One carboxyl group and five active hydroxyl groups make up chlorogenic acid. It readily reacts with free radicals and gets rid of hydroxyl radicals and superoxide anions because of its phenolic hydroxyl structure.^32^

### 3.5 Determination of the Antimicrobial Effect and Combination Test

In the combined antibiotic treatment, there are four different interactions between antibiotics. These are synergy, additive effect, differential effect, and antagonism. If the effect of the tested antimicrobials together is significantly higher than the effect of each antibiotic used alone, it is called a synergistic effect, and if it is lower, it is called an antagonistic effect. If the effect of antibiotics used in combination is the sum of their separate effects, then this is called the additive effect and is also defined as partial synergy. It was determined that there was an additive effect between VA, DA, P, OFX, and *G. fosteri* bulb water extract in all tested microorganisms. The synergistic effect was determined between OFX and extract only *S. aureus* and *P. aeruginosa* (Table 3 and Figure 4).

Chlorogenic acid, one of the common phenolic acids in plants, is known to exhibit anti-inflammatory activities.^33^ However, the mechanism of the antimicrobial effect has not yet been fully explained. It was demonstrated in a study of the antibacterial mode of action of chlorogenic acid against *Yersinia enterocolitica* that the compound may inhibit the production of biofilms and lower the established biofilm biomass of the bacterium. Additionally, it was determined that chlorogenic acid adheres to *Y. enterocolitica*, which damages bacterial cells by rupturing the cell membrane and increasing membrane permeability.^34^

### 3.6 Molecular Docking Analysis

**Figure 5:**
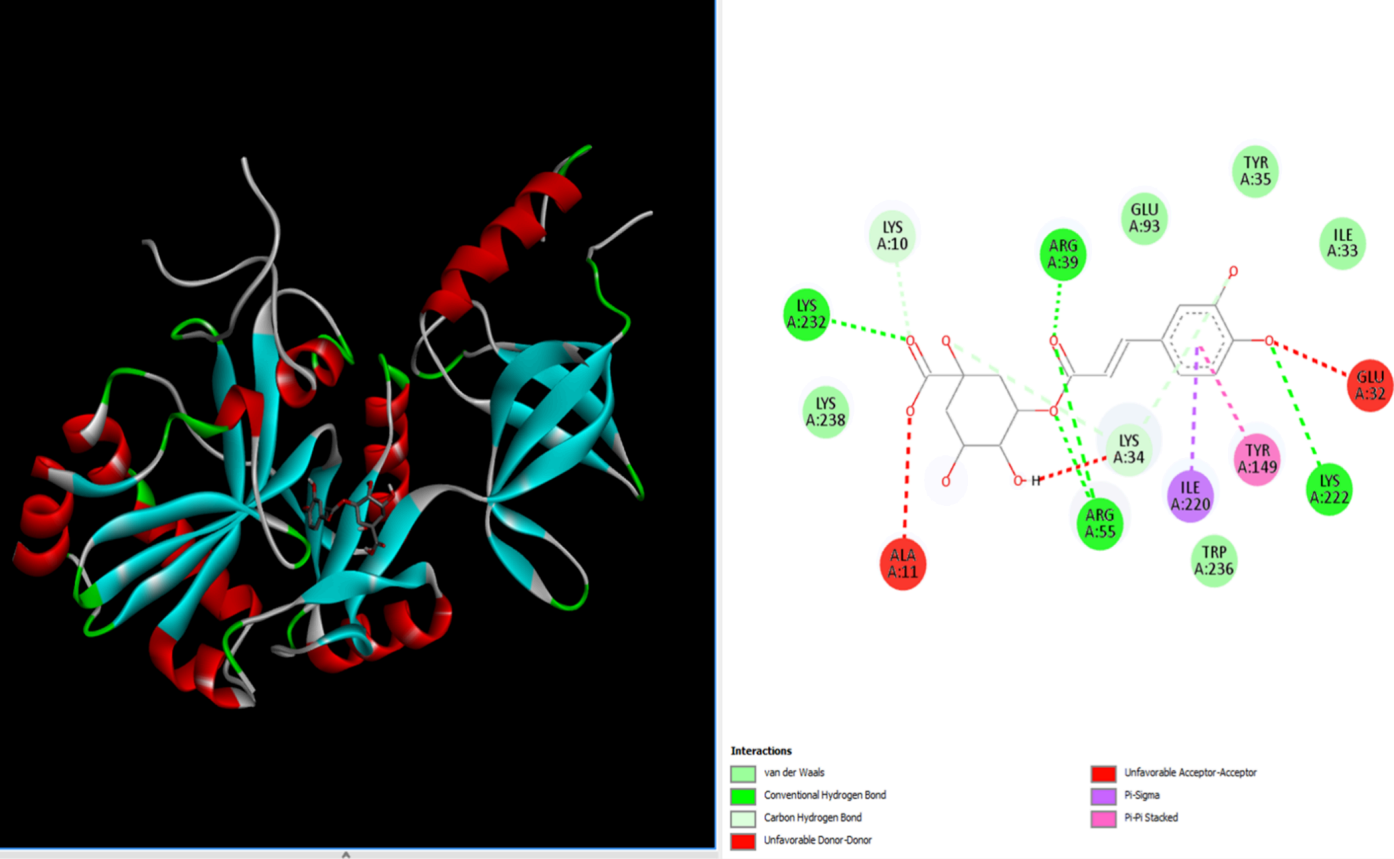
Molecular coupling between chlorogenic acid and 1A0I (DNA ligase). The image on the left shows the binding position. The image on the right shows the molecular modeling of the interaction between chlorogenic acid’s 1A0I (DNA ligase) and amino acid residues.

The binding energy resulting from the interaction of chlorogenic acid (PubChem CID: 1794427) and 1A0I (DNA ligase) (PDB 1A0I) is given in Table 4. The existence of an interaction between these two molecules was detected using the molecular docking method. The interaction between chlorogenic acid and 1A0I is shown in Figure 5.

**Table 4:** Binding Energy in the Interaction of Chlorogenic Acid (PubChem CID: 1794427) and 1A0I (DNA Ligase) (PDB ID: 1A0I)

chlorogenic acid **(1A0A)**			
mode	affinity	dist from	best mode
1	−9.1	0.000	0.000
2	−8.9	1.176	2.686
3	−8.8	1.669	2.457
4	−8.7	1.603	3.117
5	−8.4	1.435	3.182
6	−8.3	2.003	3.334
7	−7.9	1.883	3.542
8	−7.6	3.211	7.688
9	−7.0	3.810	7.977

**Figure 6:**
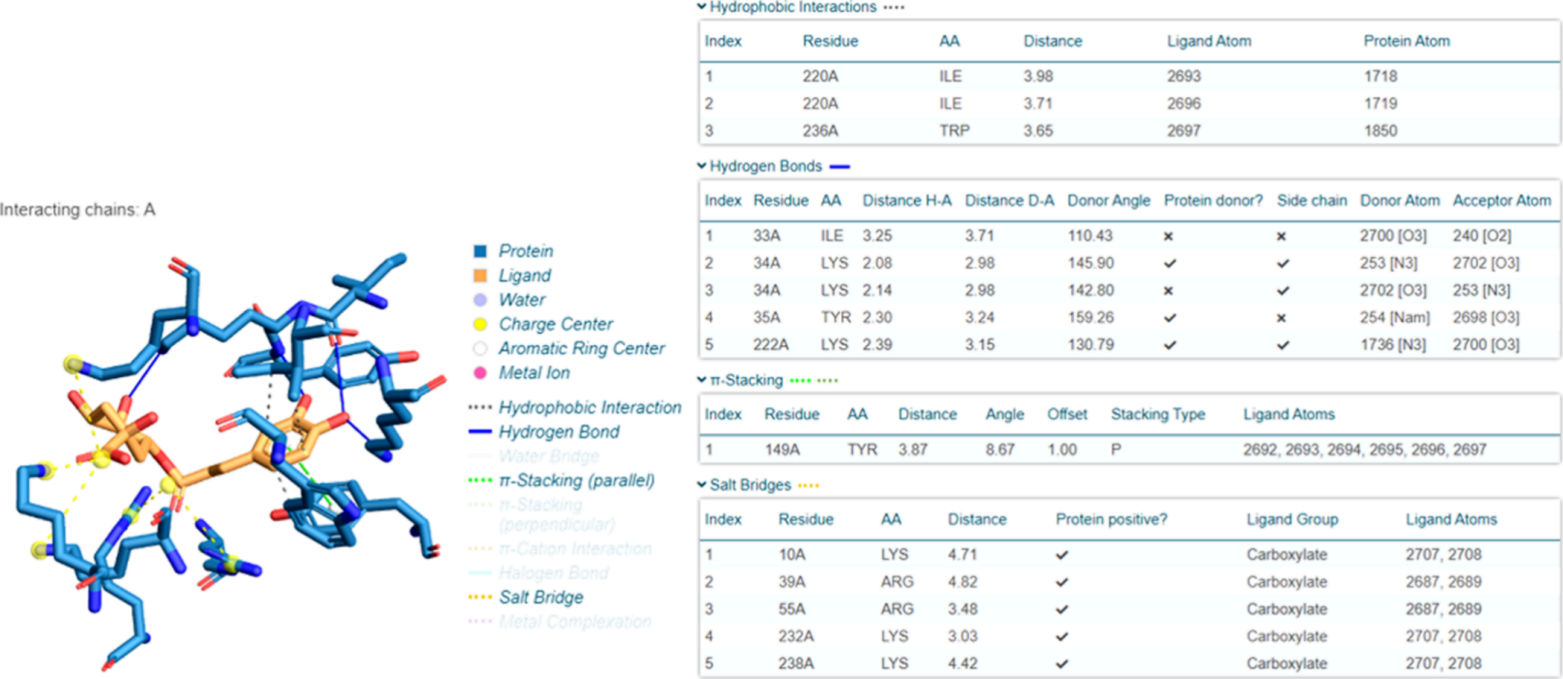
Bond lengths in molecular docking between chlorogenic acid and 1A0I (DNA ligase).

Van der Waals, conventional hydrogen bonding, and other interactions between chlorogenic acid and 1A0I have been observed (Figure 6). The van der Waals interaction was observed between residues GLU93, TYR35, ILE33, LYS222, TRP236, LYS34, ARG55, LYS238, LYS232, LYS10, and ARS39. An affinity value of −9.1 was calculated as the binding energy resulting from the interaction between the ligand and the protein. A negative value indicates that the reaction is exothermic and occurs voluntarily (Table 4). It was observed that the interaction between the crystal structure of the *S. aureus* 4G6D protein and chlorogenic acid, the primary chemical found in the flaxseed extract, inhibited the 4HI0 protein. A low energy score (−6.26841 kcal/mol) was obtained with particular residues (PRO 38, LEU 3, LYS 195, and LYS 2) from the molecular docking interaction.^35^

Numerous bacteria have been used to test the antibacterial activity of chlorogenic acid, including both Gram-positive and Gram-negative pathogens, including *Streptococcus pneumoniae* and *S. aureus*, as well as *Shigella dysenteriae*, *Salmonella typhimurium*, *K. pneumoniae*, and *P. aeruginosa*.^36,37^ According to reports, chlorogenic acid reduces the synthesis of virulence factors, inhibits the formation of biofilms, scavenges reactive oxygen species (ROS), and regulates quorum sensing in order to counter bacterial infection.^37,38^ According to Le et al., chlorogenic acid can eliminate intracellular ROS, alter lipid metabolism, and downregulate ribosomal subunits to provide its antimicrobial effects.^39^ Chlorogenic acid stress, according to metabolomic analyses, induces an intracellular metabolic imbalance of the tricarboxylic acid cycle (TCA cycle) and glycolysis, which results in metabolic instability and *Bacillus subtilis* mortality. These results contribute to our understanding of the intricate processes underlying the antimicrobial activity of chlorogenic acid and offer theoretical justification for its use as a natural antibacterial agent.^40^

## 4 Conclusions

The Amaryllidaceae family generally shows antioxidant activity,^3^ and the antioxidant property comes from the phenolic compounds found in plants. Phenolic compounds also show antimicrobial, antioxidative, and anticarcinogenic effects in the body.^41^ The antimicrobial effects of plant extracts against clinical infections are safe and effective.^42^ In this study, extracts of *G. fosteri* organs did not show significant amounts of TPC, TFC, and antioxidant properties, but leaf and stem extracts showed more antioxidant properties than bulb extracts. One of the phenolic compounds in plant leaves of caffeic acid was detected predominantly in the leaves. The DPPH inhibition values were determined to be 78 and 80% in the stem and leaf, respectively. This may be due to the galantamine found in the stem and leaves. It was determined that there was an additive effect between VA, DA, P, OFX, and *G. fosteri* bulb water extract in all tested microorganisms. The synergistic effect was determined between OFX and extracts of only *S. aureus* and *P. aeruginosa*.

The rapid performance of molecular-docking-based studies and ligand−protein interaction studies is important in terms of time and cost for new treatment strategies. In this study, the interaction of chlorogenic acid with the 1A0I protein was demonstrated for the first time. These results advance our understanding of the intricate processes underlying the antimicrobial activity of chlorogenic acid and offer theoretical justification for its use as a natural antibacterial agent.

In this study, molecular docking between chlorogenic acid and 1A0I (DNA ligase) was demonstrated for the first time, and it was thought that the antimicrobial activity of chlorogenic acid may be due to its inhibition of DNA replication.

## Notes

Research and publication ethics were complied with in the study.
